# The Stem Cell Hard Sell: Report from a Clinic's Patient Recruitment Seminar

**DOI:** 10.5966/sctm.2016-0208

**Published:** 2016-08-02

**Authors:** Paul S. Knoepfler

**Affiliations:** ^1^ Department of Cell Biology and Human Anatomy, University of California Davis School of Medicine, Davis, California, USA; ^2^ Genome Center, University of California Davis School of Medicine, Davis, California, USA; ^3^ Institute of Pediatric Regenerative Medicine, Shriners Hospital for Children Northern California, Sacramento, California, USA

## Abstract

The growing direct‐to‐consumer, stem cell clinic industry in the U.S. uses a number of strategies for patient recruitment, including self‐styled educational seminars, which may reach thousands of members of the public annually. Here I report on a first‐hand experience at such a seminar that I recently attended. Numerous specific medical claims were made at the seminar: no potential for rejection; no side effects, including no pain; proven efficacy for a variety of conditions, including in particular arthritis and pain; and U.S. Food and Drug Administration approval. I discuss the potential impact of these kinds of seminars on the public and on the stem cell field. Stem Cells Translational Medicine *2017;6:14–16*

**Significance Statement**

This article focuses on the strategies used to recruit patients via for‐profit stem cell clinic seminars and the marketing claims made as well as the impact on the public and the stem cell translational medicine field. Although the field is very aware of the existence of such seminars, this piece is significant and novel for reporting first‐hand on the specific factual details of one such seminar. Stem cell clinics conducting such self‐described stem cell educational seminars use a number of marketing strategies to recruit customers and make specific testable medical claims, which may, in some instances, misinform the public and potentially pose a threat to the stem cell field. Potential ways to address this growing challenge are discussed.

## Introduction

The American direct‐to‐consumer stem cell clinic industry has grown quickly in recent years to include at least 570 clinics 1, and there is concern that it may be based more on hype than on data 2, 3, 4, 5. It is increasingly common to see advertisements for stem cell interventions not approved by the U.S. Food and Drug Administration (FDA) 6 and for scientists to be contacted by patients considering these unproven therapies and who are seeking out additional information 7. As part of the strategy for recruiting patients, businesses marketing stem cell “treatments” have also taken to arranging gatherings that are often termed “educational seminars.” Press releases and advertisements describe these seminars as opportunities for patients to learn more about stem cells, but these seminars also appear to be an effective way for clinics to recruit new customers by making a “hard sell” to attendees. For these reasons, such meetings have informally been termed “infomercial” seminars. Reports on stem cell clinic infomercial seminars have largely been anecdotal, so they remain a poorly understood phenomenon. Here I describe my first‐hand experience attending a stem cell clinic infomercial seminar. This report includes my impressions of the strategies used by those running the seminar to sign up new customers, the marketing claims made, and the potential impact of such seminars in the broader stem cell translational medicine context.

## First Impressions

Noting an advertisement for what I perceived to be a stem cell clinic infomercial seminar, I traveled to the location to attend. To enable a higher degree of frankness in this report, I am omitting the identity of the stem cell business, the location of the seminar, and the names of the speakers. Because I am known in the stem cell clinic world, I was uncertain as to the reception I might receive. I strove to be unobtrusive when I arrived, but soon after I sat down in the audience at the hotel meeting room where the seminar was about to begin shortly, one of the organizers came up to me and indicated that he knew who I was.

My purpose was to gather information about this clinic and experience first‐hand what a stem cell clinic infomercial seminar was like. My sense was that they knew I was not there as a potential customer. Those running the seminar frequently sat or stood immediately behind me during the meeting, perhaps as an attempt at intimidation or to see the notes I was taking. Because I was identified by the people running the seminar prior to its beginning, a caveat in this piece is that they could have changed aspects of the seminar because they knew I was in the audience. However, I saw no clear evidence of that.

My initial overall observation of the seminar environment was striking and disconcerting. There were approximately 30 people in attendance, and most were senior citizens. My impression was that many of the attendees had serious medical conditions, which sparked concern in my mind that they could be vulnerable to a sales pitch. There were quite a few couples in the audience.

The seminar team (mostly dressed in medical scrubs) handed out a clipboard to each attendee with three pieces of paper very much like those that one might be given on arriving at a new doctor's office. We were told to fill in our information and then to return the sheets back to them before we left. Notably, the top sheet was a credit application. On another page, the clinic asked for extensive personal information, including name, age, birthdate, address, e‐mail, and phone number. Additional questions asked about medical conditions, as well as medical tests that had already been conducted.

As the seminar began, the audience was told to hold all questions until after the speakers were done, and then they would talk one‐on‐one with attendees. I had hoped to ask a few questions from my place in the audience, which others present could hear, as a means to catalyze a meaningful discussion of key issues, but this “rule” seemed to negate that chance, and I had no interest in being disruptive of the meeting.

## Making It Personal

The speaker introduced himself as a veteran of the stem cell field. I will call him by the pseudonym Ted. He discussed his experience with a successful stem cell business in another state and portrayed himself as a stem cell expert. Fairly quickly he began an anecdote about his own family members. Ted reported that a family member had suffered a major illness and that as a result he knew what it meant to be facing a difficult medical situation in the family. He then did a survey of the audience via raised hands to ask about various medical conditions that brought them there. The most common condition that brought people to the seminar was arthritis and the associated pain.

Ted next asked the audience to close their eyes and ask themselves, “What could you do in the past that you can't do now?” With my eyes open, I estimated that nearly every member of the audience had closed his or her eyes. I wondered about the impact of this approach by the seminar organizers on the audience's state of mind. Ted went on to speak about how the U.S. health care system is broken before starting in on how wonderful stem cells are as a form of medicine.

## Questionable Medical Claims

It was at this point that Ted began a more intense sales pitch focused on this particular clinic's marketed kind of stem cells, amniotic stem cells, which is the main basis for their “treatments,” although adipose stem cells were also mentioned briefly. He used the analogy of stem cells as that uncle or other relative we all have who is a “Mr. Fixit,” who can “fix anything.” I interpreted this as a potential medical claim, the first in a series presented (Table 1), meaning that their stem cells can successfully treat many conditions. At no time during the meeting did I note disclaimers or waivers of any kind regarding this or other claims. He described the different kinds of stem cells, reporting incorrectly that it is illegal to use or even study embryonic stem cells. He described the amniotic cells this clinic would transplant into patients as being akin to a “new car” and having no potential for rejection once transplanted. This statement of no possible immunorejection was a second, concrete medical claim made to the audience, and it was made with no conditions or qualifiers.

**Table 1:** Summary of stem cell seminar marketing claims

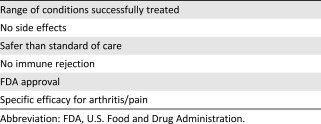

The treatment was then described in further detail, leading to additional medical claims. For instance, Ted indicated that the treatment would be a same‐day, painless injection, so patients would just walk out with a bandage. “There is no surgery and this is the new gold standard,” he said. Furthermore, he reported that they have observed “no side effects,” another unambiguous medical claim. In contrast, traditional procedures such as knee replacements have “many risks,” we were told, including heart attacks. Ted claimed to the seminar audience that 5,000 people die each year from a heart attack after knee surgery. I fact‐checked this claim and found a recent study indicating no major long‐term increase in the rate of heart attacks following knee surgery 8. Thus, the clinic statement, rather than being based on data to accurately inform the seminar audience, was rather a claim most likely intended to make the treatment being sold at the seminar appear dramatically safer and more effective than the standard of care, such as joint replacement surgery.

He made another definite, quantitative medical claim: “90% of patients had a 50% or better improvement” with this treatment. Ted went on to try to bolster the claim of efficacy with before‐and‐after photos from patient x‐rays and MRIs. He even showed data from a competing clinic in another state. He flashed a slide with a table of numbers of their own data purporting to show great improvements in patients’ perception of pain after receiving the stem cell “treatment.” He invoked some specific patient testimonials, including a video, and he also made a claim that the clinic had treated a particular professional sports celebrity with stem cells.

## Closing the Deal

Ted then indicated that all the audience members should close their eyes again. He told the attendees to visualize and project themselves 6–12 months in the future. “Put yourself in the future where now you can do those things that you couldn't before.” I saw a few audience members nodding in agreement. Then Ted said, “It is your choice to be in pain or not.” My sense was that this had a powerful impact on the audience.

During the remaining few minutes, Ted made an additional medical claim of FDA approval: “While this treatment is FDA‐approved, it is not covered by insurance.” However, it is not clear to me, as someone who has been following the stem cell field for many years, that the treatment that was being sold at this meeting was actually FDA‐approved. My impression, on the basis of all that Ted and the other speaker said, was that the “stem cells” being marketed to the audience were living amniotic stem cells, but using living amniotic stem cells for treatment of nonhomologous medical conditions such as arthritis could squarely place a treatment of this kind into the realm of an unapproved biological drug. In this sense, such a product would not be “FDA‐approved.” Alternatively, if the product in question is an extract of amniotic cells with no living cells, then—to me at least—this conflicted with how the product was being portrayed to the audience, which again strongly implied the use of living stem cells. More practically speaking, what the second part of this statement from Ted meant was that the patients would have to pay somehow because insurance would not.

The price of the treatment was quoted as $5,999, but we were told that there was a “seminar special” under way during which attendees could get $1,000 off the price. However, this deal was only good “if you sign up today at the seminar.” This felt to me like a hard sell, and I turned back to the credit application on my clipboard, wondering about patients going into debt. A number of people had been filling out some of the forms during the seminar. Finally, Ted added that monthly payment plans below $100 were possible as well, and he said rather generally that many people were signing up, as reflected by the fact that the clinic was already booked 1–2 months out.

## Conclusion

Overall, this experience felt more like attending a persuasive entertainment show or something on a television shopping network than an educational seminar. While I do not know what other stem cell clinic infomercial seminars are like first‐hand, my sense from watching clinic marketing videos, including those on YouTube, is that the one I attended was fairly representative of what goes on at such customer recruitment events.

Approximately 30 people attended the one clinic seminar at which I was present, and, on the basis of an informal observation of advertisements, I have noted that the sponsors have held many of these seminars. Thus, it is possible that approximately 200 people have seen just this one type of seminar alone in one city in the U.S. from a single clinic. Given the growing realization that there are hundreds of stem cell clinics in the U.S. today in 2016 and the observation that many hold recruitment seminars, it is possible that thousands of members of the public each year are attending infomercial seminars that provide misleading or even outright factually incorrect information about stem cells and questionable medical claims. This could not only lead many patients to receive unnecessary, unsafe, or ineffectual treatments, but it may also contribute to public confusion about stem cells and the field of stem cell clinical research. These seminars represent only one type of an assortment of recruitment methods, including Internet, radio, newspaper, and television ads for various clinics, which may also contain dubious statements. I believe that such stem cell clinic marketing poses a significant threat to public perception and understanding of the legitimate stem cell translational medicine field.

Although individual stem cell scientists such as myself can attend such meetings, ask questions, and aim to provide correct information on stem cells, my feeling is that such efforts alone will be insufficient to counter the negative impact from this phenomenon. The FDA has thus far not been particularly effective in managing the mushrooming direct‐to‐consumer stem cell industry in the U.S. What may prove more effective is contacting the Federal Trade Commission to file complaints about misleading advertising. Another potentially effective strategy is for stem cell and regenerative medicine organizations to take more assertive action in educating the public to counter the negative effects of stem cell infomercial seminars. This growing phenomenon must not be ignored, and at least part of the solution will come from actions taken by stem cell researchers and their organizations.

## Disclosure of Potential Conflicts of Interest

The author indicated no potential conflicts of interest.
